# Genotype-Driven Diagnosis Enables Targeted Pharmacological Treatment in Brunner Syndrome: A Novel Splice-Site *MAOA* Variant and Case-Based Review

**DOI:** 10.3390/ijms27146223

**Published:** 2026-07-12

**Authors:** Elisa Gravagno, Melissa Bellini, Enrico Ambrosini, Anita Luberto, Sabrina Busciglio, Giulia Vitetta, Ilenia Rita Cannizzaro, Antonietta Taiani, Valeria Barili, Antonio Percesepe, Vera Uliana, Davide Martorana

**Affiliations:** 1 Medical Genetics Unit, University Hospital of Parma, 43126 Parma, Italy; 2 Pediatrics and Neonatology Unit, Guglielmo da Saliceto Hospital, 29121 Piacenza, Italy; 3 Medical Genetics, Department of Medicine and Surgery, University of Parma, 43126 Parma, Italy

**Keywords:** Brunner syndrome, *MAOA*, splice-site variant, monoamine dysregulation, genotype-driven therapy, serotonin antagonist and reuptake inhibitor, SARI, clinical exome sequencing

## Abstract

Brunner syndrome is a rare X-linked neurodevelopmental disorder caused by loss-of-function (LOF) variants in the monoamine oxidase A gene (*MAOA*), which encodes monoamine oxidase A, a key enzyme involved in the degradation of monoamine neurotransmitters such as serotonin, norepinephrine, and epinephrine. Impaired MAOA activity leads to abnormal monoamine accumulation and disruption of monoaminergic signalling, resulting in intellectual disability and behavioural dysregulation. Here, we systematically summarize the molecular landscape and report a genotype-driven diagnosis of MAOA deficiency in a patient presenting with intellectual disability and no reported family history. Clinical exome sequencing (cES) identified a novel splice-site variant in the *MAOA* gene that had not been detected by first-line diagnostic approaches. Functional analysis of patient-derived mRNA demonstrated intron 8 retention leading to a premature stop codon, consistent with a LOF mechanism. Based on the molecular diagnosis, the patient received treatment with serotonin antagonist and reuptake inhibitor (SARI) class medication, which was associated with improvement in social behaviour and sleep disturbances. Notably, to the best of our knowledge, this represents the first reported use of SARI therapy in MAOA deficiency. Although SARI therapy in this condition remains off-label, this observation provides preliminary evidence suggesting a potential therapeutic benefit. Our findings expand the mutational spectrum of the *MAOA* gene and highlight the importance of molecular diagnosis driving personalized management in rare neurogenetic disorders.

## 1. Introduction

Neurodevelopmental disorders (NDDs) are a heterogeneous spectrum of conditions with onset in childhood that include intellectual disability, autism spectrum disorder, and rare monogenic syndromes [1]. In recent years, the widespread implementation of next-generation sequencing (NGS) has markedly increased the diagnostic yield of these disorders, enabling the identification of pathogenic variants in genes critical for brain development and neurotransmitter regulation [2]. Beyond providing an etiological diagnosis, molecular characterization is increasingly relevant for patient management, genetic counselling and the implementation of targeted therapeutic strategies with a precision medicine framework [3]. In rare NDD, genotype-driven diagnosis is increasingly proven as a clinically actionable step, as it may shorten the diagnostic trajectory, refine prognosis, guide surveillance, and support individualized therapeutic decisions [2,4]. This approach is particularly relevant for disorders affecting neurotransmitter metabolism, in which the identification of the causative molecular defect may provide mechanistic insights and generate testable hypotheses for targeted pharmacological intervention [5]. Among rare monogenic disorders affecting behavioural and cognitive function, monoamine oxidase A (MAOA) deficiency—also known as Brunner syndrome (OMIM #300615)—is a paradigmatic example of a neurotransmitter metabolism disorder with direct clinical implications. First described in 1993 in a large Dutch pedigree, this X-linked recessive condition is characterized by variable intellectual disability and prominent behavioural dysregulation, including impulsive aggression, resulting from impaired monoamine degradation [6]. Despite its historical and biological relevance, only a limited number of cases have been reported to date, and the genetic, clinical, and therapeutic spectrum of the disorder remains incompletely delineated. Nevertheless, the few reported genotype-informed therapeutic attempts suggest that molecular diagnosis may have practical implications for patient management: in selected individuals carrying functionally disruptive *MAOA* variants, serotonin reuptake inhibitors combined with dietary tyramine restriction were associated with behavioural improvement and normalization or partial correction of monoamine-related biochemical abnormalities [7,8,9,10].

Here, we provide a systematic summary of the molecular landscape reported in clinical cases, genotype–phenotype correlations, and available therapeutic approaches. In addition, we describe a previously unreported splice-site variant in the *MAOA* gene, expanding the mutational spectrum of the disorder and illustrating how molecular diagnosis can directly guide targeted pharmacological management and improve clinical outcomes.

## 2. Results

### 2.1. MAOA Loss-of-Function Variants Predominate Among Clinically Reported Cases

**Figure 1:**
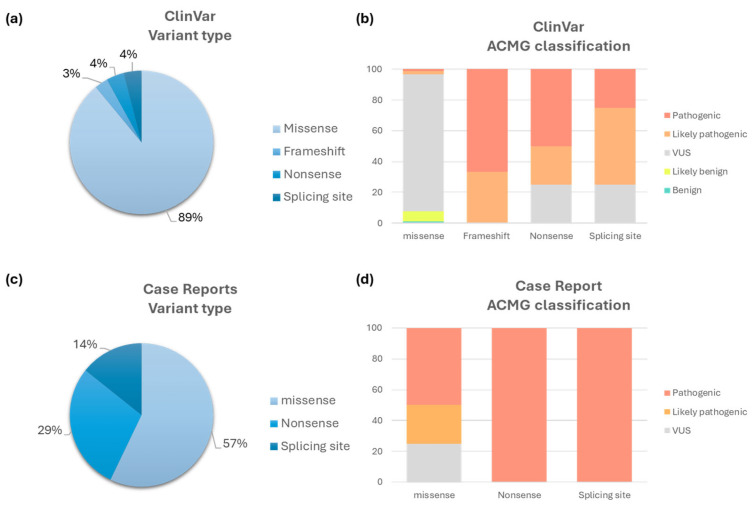
Distribution and ACMG classification of *MAOA* variants from ClinVar dataset and published Brunner syndrome cases: (**a**) Molecular distribution of *MAOA* variants reported in the ClinVar database (*n* = 100); (**b**) pathogenicity classification of ClinVar alterations, according to ACMG criteria, highlighting a higher proportion of pathogenic and likely pathogenic variants among frameshift, nonsense, and splice-site variants compared to missense variants; (**c**) molecular distribution of variants in published case reports; (**d**) ACMG-based pathogenicity classification of variants reported in case studies, where the majority are classified as pathogenic or likely pathogenic. MAOA, monoamine oxidase A; ACMG, American College of Medical Genetics and Genomics; VUS, variant of uncertain significance.

The search in the ClinVar database identified a total of 142 variants (Supplementary Table S1), of which 100 were located within the coding regions of the *MAOA* gene, providing an overview of the mutational spectrum of *MAOA*. Missense variants accounted for most of the reported alterations (Figure 1a), indicating a strong bias toward single amino acid substitutions. However, only a limited number of missense changes are classified as pathogenic or likely pathogenic, with a substantial proportion of variants of uncertain significance (VUS) (Figure 1b). In contrast, loss-of-function alterations, including frameshift, nonsense, and splice-site variants, showed a higher proportion of pathogenic or likely pathogenic classifications (Figure 1b), consistent with their predicted disruptive impact on protein function. The literature search retrieved a total of 24 relevant articles in PubMed. The earliest report, published in 1993, described the first family affected by MAOA deficiency; this condition was subsequently named Brunner syndrome, a term that has been widely adopted in the medical literature [7]. All selected articles were reviewed, summarizing the clinical, genetic, and biochemical features associated with this condition (Table 1). Within the curated set of published case reports, missense variants were the most frequently observed, with a smaller proportion of nonsense and splice-site variants (Figure 1c). Notably, in contrast to the ClinVar dataset, the majority of variants described in case studies were classified as pathogenic or likely pathogenic (Figure 1d). This discrepancy likely reflects a reporting bias toward clinically significant cases, as variants described in the literature are typically identified in the context of a defined phenotype. Collectively, these findings highlight the distinct molecular distributions and pathogenicity classifications between genomic variants databases and published clinical case reports, emphasizing the importance of integrating both sources for a comprehensive interpretation of the *MAOA* mutational landscape.

**Figure 2:**
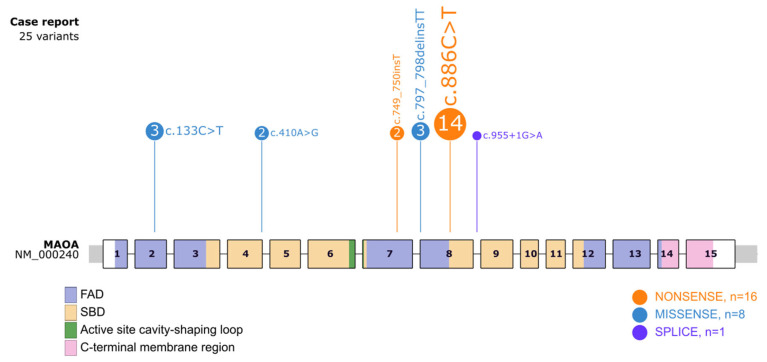
*MAOA* variant distribution as described in case reports affected by Brunner syndrome: Schematic representation of the *MAOA* gene showing localization of variants identified in the published Brunner syndrome cases across exons 1–15 (represented as rectangles). Variants are colour-coded according to the molecular change, with nonsense in orange, missense in blue, and splice junction variants in purple, and the number of recurrent variants is indicated within each circle. Distinct functional regions of the MAOA protein are shown below the gene structure using different colours: violet indicates the flavin adenine dinucleotide (FAD)-binding domains (residues 13–88, 220–294, and 400–462); orange marks the substrate-binding domains/SBD (residues 89–219 and 295–399); green represents the active-site cavity-shaping loop (residues 463–506); and pink denoted the C-terminal membrane region (residues 463–506).

The distribution of *MAOA* variants reported in case studies is illustrated in Figure 2, highlighting both their positional clustering along the gene and their molecular annotation. A total of 25 variants were mapped across the coding sequence, with a clear predominance of LOF alterations, particularly nonsense variants (*n* = 16), followed by missense (*n* = 8) and a single splice-site variant (*n* = 1, current case). These variants are distributed across multiple exons, with a noticeable enrichment in the central region of the gene, including exons encoding functionally relevant domains such as the flavin adenine dinucleotide (FAD)-binding and substrate-binding domains. Notably, several recurrent or proximal variants were observed within exons 7 and 8, suggesting potential mutational hotspots or regions of increased functional constraint. The localization of variants within key structural domains further supports their likely impact on enzymatic activity, which is consistent with the high proportion of pathogenic or likely pathogenic classifications reported in case studies. Overall, this distribution underscores the relevance of variant position and type in shaping the clinical presentation of MAOA deficiency.

In this context, we report a novel splice-site variant in the *MAOA* gene identified in a patient with intellectual disability. Conventional diagnostic approaches were unable to determine the genetic cause of the disorder. However, clinical exome sequencing (cES) enabled the identification of the causative pathogenic variants, allowing the establishment of a precise molecular diagnosis and supporting precision medicine-oriented clinical management.

### 2.2. Clinical Exome Sequencing Identified a Novel Pathogenic Splice-Site Variant Enabling Targeted Therapy

The proband is a 24-year-old male presenting with a history of developmental delay and mild intellectual disability consistent with a neurodevelopmental disorder.

Neonatal cranial ultrasound revealed asymmetry of the lateral ventricles (left > right), which remained stable on follow-up, while cardiac ultrasound and EEG were unremarkable. Development was characterized by global delay, with late acquisition of language (first words at 27 months, first phrases at 39 months), delayed independent walking (at 20 months), and difficulties in coordination and fine motor skills. Relative macrocephaly and a negative growth trend were also noted.

Genetic testing performed at 3 years of age, including karyotype, Fragile X analysis, and subtelomeric rearrangement testing, yielded normal results. Neuropsychiatric evaluation documented a moderate intellectual disability (IQ 65), associated with impairments in language, social communication, and behavioural regulation.

The clinical picture was further characterized by autistic features, including reduced eye contact in early infancy, preference for solitary play, restricted interests, stereotyped motor behaviours, and atypical sensory responses.

Over time, behavioural assessment revealed the presence of phobic traits without evidence of overt aggressive or impulsive behaviour. Sleep disturbances were prominent, characterized by recurrent night terrors and parasomnia, which have persisted over time. No specific serotonergic symptoms were reported, and biochemical investigations did not reveal detectable alterations.

**Figure 3:**
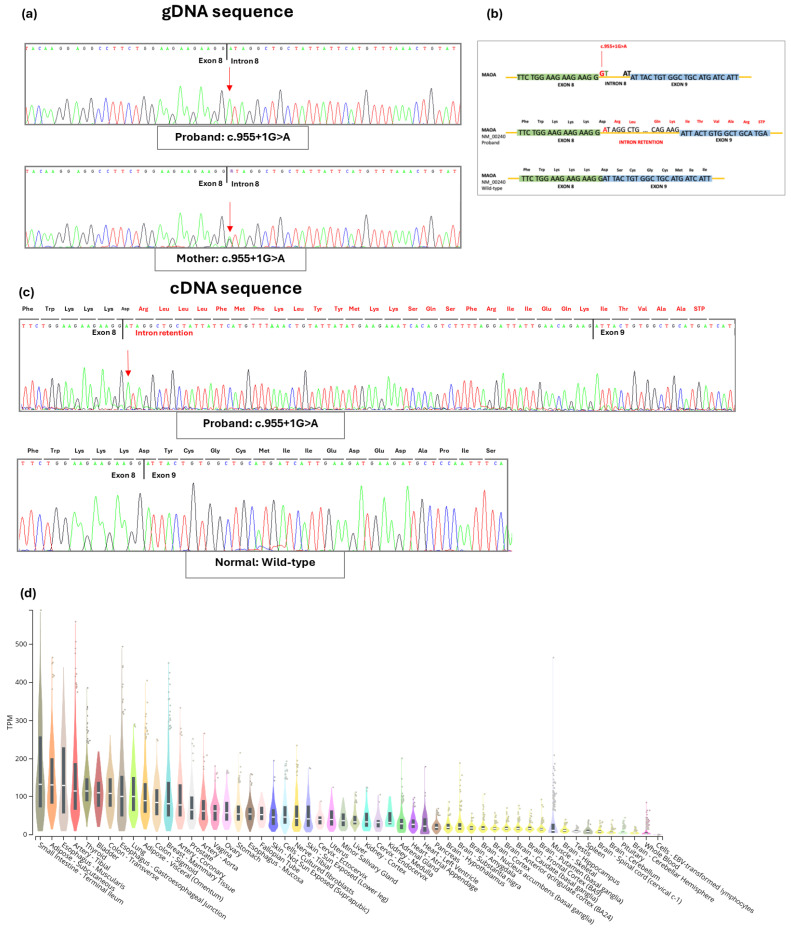
Molecular characterization of the *MAOA* c.955+1G>A splice-site variant and *MAOA* tissue gene expression. (**a**) Sanger sequencing electropherograms showing the hemizygous c.955+1G>A variant in the proband and heterozygous state in the mother. The arrow indicates the site of c.955+1G>A variant; (**b**) schematic representation of the *MAOA* exon–intron structure around exon 8, illustrating the disruption of the canonical donor splice site and the predicted activation of a cryptic splice site within intron 8; (**c**) cDNA Sanger sequencing electropherograms demonstrating aberrant splicing in the proband, with retention of 73 bp of intron 8, resulting in a frameshift and premature stop codon, compared to the normal transcript observed in the control. The arrow indicates the site of the variant; (**d**) tissue-specific expression profile of *MAOA* based on the Genotype-Tissue Expression (GTEx) data, showing high expression levels in brain regions. Scale is expressed in transcripts per million (TPM).

A trio clinical ES (cES) was performed and allowed the identification of a hemizygous splice-site variant in *MAOA* (NM_000240.4:c.955+1G>A, GRCh37/hg19), located in the canonical donor splice site of exon 8. The variant has not been previously reported and is absent from the gnomAD population database (v2.1). Sanger sequencing confirmed the presence of the variant in the proband and demonstrated maternal inheritance (Figure 3a). According to ACMG criteria, the variant was classified as likely pathogenic (PVS1, very strong; PM2, moderate), consistent with a predicted LOF mechanism. In order to assess the functional impact on the splicing mechanism, RT-PCR amplification of the *MAOA* transcript spanning exons 7 to 10 was performed using blood-derived cDNA from the proband and a healthy control. In silico prediction with the SpliceAI tool indicated a marked disruption of the canonical donor splice site (DS_DL = 0.96) and the activation of a downstream cryptic donor site located 73 bp within intron 8 (DS_DG = 0.34). Experimental validation confirmed aberrant splicing, with splice donor site loss of exon 8 and retention of 73 bp of intron 8 sequence in the mature transcript (Figure 3b,c). This alteration introduces a premature stop codon, consistent with a truncated protein and a predicted degradation via nonsense-mediated decay (NMD), as the putative stop codon lies upstream of the final exon–exon junction. Expression data from the Genotype Tissue Expression (GTEx) dataset indicate that *MAOA* is highly expressed in brain tissues (Figure 3d), supporting the relevance of LOF variants in NDD phenotypes. Segregation analysis further identified the same variant in the heterozygous state in the proband’s sister, consistent with X-linked inheritance. Taken together, the molecular and functional evidence supports the LOF effect of the *MAOA* c.955+1G>A variant, consistent with impaired monoamine degradation.

**Figure 4:**
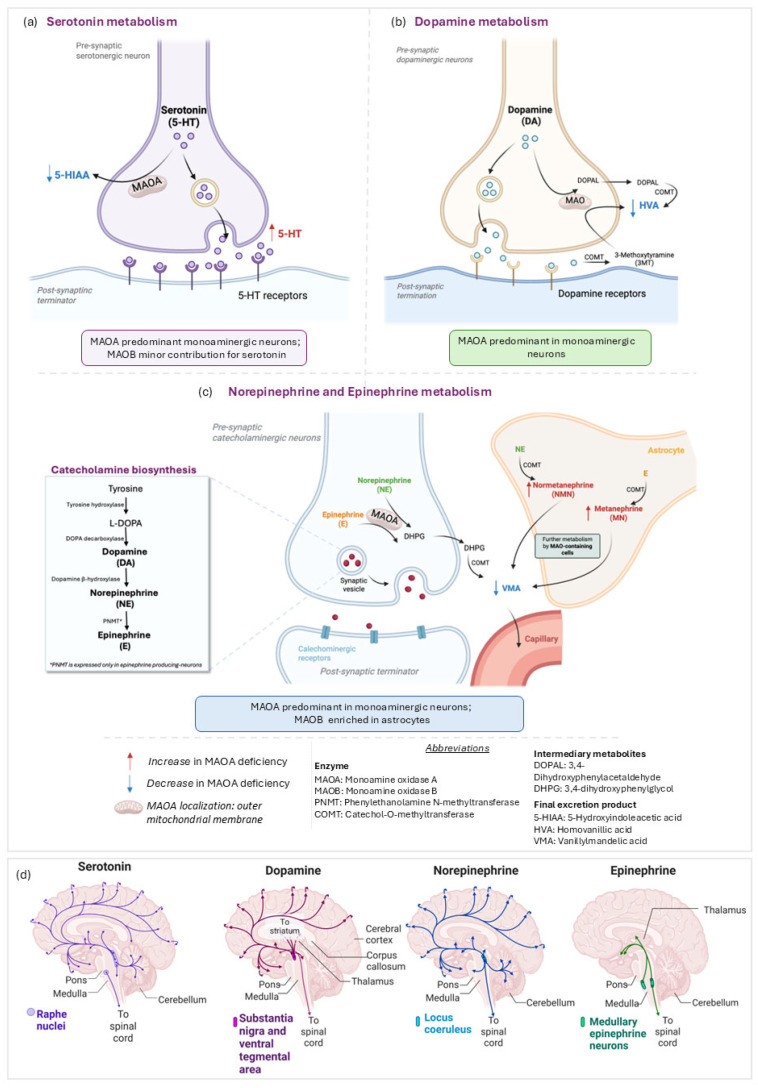
Schematic representation of serotonin, dopamine, norepinephrine, and epinephrine metabolism, highlighting the role of MAOA and the metabolic profile associated with MAOA deficiency: (**a**) Serotonin metabolism in serotonergic neurons. Serotonin, also referred to as 5-hydroxytryptamine (5-HT), is preferentially degraded through MAOA-mediated oxidative deamination, followed by downstream conversion to 5-hydroxyindoleacetic acid (5-HIAA), excreted in urine. (**b**) Dopamine (DA) metabolism in dopaminergic neurons. Dopamine can be metabolized by MAOA to 3,4-dihydroxyphenylacetaldehyde (DOPAL), which is further converted through aldehyde dehydrogenase-dependent and catechol-O-methyltransferase (COMT)-dependent reactions to homovanillic acid (HVA). (**c**) Biosynthesis, storage, release, and metabolism of norepinephrine (NE) and epinephrine (E) in catecholaminergic synapses. MAOA predominantly contributes to intraneuronal oxidative deamination of catecholamines at the outer mitochondrial membrane, while COMT, expressed in extraneuronal cells including astrocytes, catalyzes O-methylation reactions. These combined pathways generate intermediate metabolites such as normetanephrine (NMN), metanephrine (MN), and 3,4-dihydroxyphenylglycol (DHPG), ultimately contributing to vanillylmandelic acid (VMA) formation. (**d**) Major anatomical distribution of serotonin-, dopamine-, norepinephrine-, and epinephrine-producing pathways in the human brain. In MAOA deficiency, impaired oxidative deamination results in the accumulation of serotonin and intermediary metabolites including normetanephrine (NMN) and metanephrine (MN), whereas the concentrations of the final metabolites such as 5-HIAA, HVA, and VMA are typically reduced (red and blue arrows, respectively). Overall, this schematic highlights the compartmentalized organization of monoamine metabolism in the central nervous system and the predominant role of MAOA in neuronal serotonin, dopamine, norepinephrine, and epinephrine metabolism.

Given the central role of MAOA in serotonin metabolism (Figure 4) and its high expression in brain tissues, these findings provided a rationale for a targeted pharmacological approach aimed at modulating serotonergic signalling rather than further increasing synaptic serotonin levels. Accordingly, the proband was treated with Trazodone, a serotonin antagonist and reuptake inhibitor (SARI), administered at a dose of 75 mg in the evening. Prior exposure to selective serotonin reuptake inhibitors (SSRIs) had resulted in increased anxiety, prompting the switch to SARI-based therapy. Under this regimen, a global clinical improvement was reported, including enhanced cognitive performance, better social interaction, increased attention, and improved relationships with peers and adults. However, the treatment did not substantially impact sleep-related disturbances, as night terrors, phobic symptoms, and nocturnal muscle spasms persisted, despite a general improvement in sleep quality.

Family history revealed that the mother experienced mild depression, successfully managed with SSRIs, while the sister did not present mood disorders, although mild insecurity traits were noted. Importantly, neither the proband nor family members exhibited clear serotonergic features.

## 3. Discussion

Brunner syndrome is a rare X-linked recessive disorder characterized by variable intellectual disability and impulsive aggression, accompanied by biochemical alterations resulting from impaired MAOA activity [4]. MAOA is one of two primary isoenzymes (MAOA and MAOB) belonging to the monoamine oxidase family and is expressed in the outer mitochondrial membrane. MAOA and MAOB catalyze the oxidative deamination of dietary and biogenic amines. Monoamine neurotransmitters play a central role in regulating brain functions such as mood, motivation, cognition, motor control, and physiological processes including stress response and sleep [5,6,7,11]. MAOA preferentially leads to the oxidative deamination of serotonin (5-hydroxytryptamine, 5-HT), norepinephrine (NE), and epinephrine (E). Loss of MAOA activity leads to impaired degradation of key monoamine neurotransmitters, causing their abnormal accumulation and disrupting monoaminergic signalling pathways (Figure 4) [8,9]. Structural studies of monoamine oxidases have investigated the three-dimensional organization and catalytic mechanisms of MAOA, underlining substrate specificity and inhibitor-binding properties [12]. These insights provide a framework for interpreting the functional consequences of pathogenic *MAOA* variants. Experimental models further clarify the pathophysiology of MAOA deficiency: *MAOA* knockout mice exhibit heightened aggression toward both familiar and unfamiliar conspecifics, reduced anxiety-like behaviour, and impaired stress regulation, thereby reproducing core neurocognitive and behavioural abnormalities associated with Brunner syndrome [13]. Case reports of Brunner syndrome remain limited but consistently describe affected individuals carrying nonsense or missense variants in the *MAOA* gene, underlining the significant impact of amino acid substitutions on MAOA enzyme efficiency (Table 1). Notably, reported variants induce notable changes in electrostatic interactions between the reacting moiety and its enzymatic environment, resulting in decreased catalytic performance of MAOA [14,15].

Clinical management of patients with MAOA deficiency has primarily been explored in preclinical studies investigating the potential therapeutic effects of selective serotonin reuptake inhibitors (SSRIs) [16]. In *MAOA* knockout mouse models, SSRI administration reduces aggressive behaviours, decreases perseverative responses, and improves social deficits, suggesting a possible therapeutic benefit in this condition. The first clinical report describing the management of affected individuals was provided by Palmer et al. [9], who treated a family (a mother and her two sons) with SSRIs in combination with dietary modification, specifically the elimination of tyramine-containing foods. This approach resulted in significant clinical improvement, including a reduction in behavioural symptoms and normalization of biochemical parameters associated with monoamine dysregulation. However, the use of SSRIs in MAOA deficiency appears paradoxical. SSRIs increase synaptic serotonin levels through inhibition of the serotonin transporter (SERT), whereas LOF variants in *MAOA* are already associated with elevated serotonin concentration. One proposed explanation for the observed clinical benefit is a reduction in peripheral serotonin levels due to inhibition of platelet serotonin transporters, considering that platelets constitute the main storage site for serotonin in the bloodstream. More recently, a carrier mother presenting with mild intellectual disability, palpitations, headache, abdominal pain, and swelling was treated with SSRIs and showed partial symptomatic improvement [10].

In this context, we report a genotype-driven diagnosis in a patient with intellectual disability and no reported family history. cES enabled the identification of a novel splice variant in the *MAOA* gene that had been missed by first-line methods for diagnosing intellectual disability. Sanger sequencing confirmed the maternal origin. Functional characterization of this variant using the patient’s mRNA demonstrated intron 8 retention and the introduction of a premature stop codon, resulting in early protein truncation. This alteration is predicted to trigger NMD, leading to *MAOA* LOF. Segregation analysis was extended to the proband’s asymptomatic sister, who carries the familial variant. Clinical re-evaluation of the mother enabled the identification of previously unrecognized clinical features consistent with MAOA deficiency, including moderate depression [17] and headaches [9,10], which have been reported to be associated with MAOA dysfunction. The proband’s sister did not exhibit any clinically significant mood disorders; however, she was described as having mild insecurity traits characterized by reduced self-confidence and a tendency toward social hesitation, in the absence of significant anxiety or functional impairment. The identification of the *MAOA* variant provided a rationale for a targeted pharmacological intervention using Trazodone, an antidepressant belonging to the class of serotonin antagonist and reuptake inhibitor (SARI) [18]. Trazodone exhibits a multimodal mechanism of action, combining serotonin reuptake inhibition with antagonism at postsynaptic 5-HT subtypes (5-HT_2_A/2C receptors), as well as modulatory effects on adrenergic (α1) and histaminergic (H1) receptors. This pharmacodynamic profile may be particularly relevant in the context of MAOA deficiency, where dysregulated serotonergic and catecholaminergic signalling could underlie behavioural disturbances, sleep dysregulation, and affective symptoms. Although Trazodone is approved for the treatment of major depressive disorder and is widely used off-label for insomnia and anxiety [19], its application in patients with MAOA deficiency has not been previously reported. Clinical studies have shown that Trazodone has efficacy comparable to SSRIs, with a distinct tolerability and pharmacological profile [20]. Although the molecular diagnosis provided a rationale for targeting monoaminergic signalling, no detectable biochemical abnormalities were identified in the current patient. Therefore, the use of trazodone should be interpreted as an exploratory, genotype-based therapeutic attempt rather than as a treatment directly guided by patient-specific biochemical evidence.

Proband treatment with Trazodone was associated with an improvement in social behaviour and sleep perturbation. This novel therapeutic strategy differs from previously reported SSRI-based treatment, and our observed clinical response suggests that SARI may expand the existing repertoire of pharmacological treatment options for disorders associated with impaired monoamine metabolism. Further studies are warranted to clarify the mechanistic basis and generalizability of this approach. Although the use of SARI in MAOA deficiency remains off-label (as SSRI-based treatment) and their mechanisms of action in this context are not fully understood, our observation adds to the limited clinical evidence suggesting a potential therapeutic benefit. More broadly, this case highlights the clinical relevance of establishing a precise molecular diagnosis in rare NDD, as it may directly inform patient management and support the implementation of personalized therapeutic strategies.

## 4. Materials and Methods

### 4.1. Review

Variants were retrieved from the ClinVar database using the following filtering criteria: germline classification (benign, likely benign, variant of uncertain significance [VUS], likely pathogenic, and pathogenic), molecular consequence (missense, frameshift, nonsense, and splice-site variants), and variant size (short variants < 50 bp). ClinVar variants with summary evaluations and individual submitter annotations were retrieved from the 10 March 2026 XML file. Variants located within the 5′-UTR (untranslated region) regions were excluded (Supplementary Table S1—*MAOA* variants dataset).

A systematic literature search was conducted in PubMed to identify studies related to monoamine oxidase A (MAOA) deficiency. The search was performed using the keywords “MAOA” AND “Brunner”. Publications available up to April 2026 were considered.

### 4.2. New Patient Inclusion

In compliance with the local ethical guidelines and the Declaration of Helsinki, the new patients included in this study provided informed consent for genetic analysis and results publication. Ethical review and approval were waived for this study according to the local policy; informed consent is considered sufficient for reports of an observational nature concerning a limited number of patients.

### 4.3. Clinical Exome Sequencing

Genomic DNA was isolated from circulating lymphocytes collected in a peripheral blood sample. cES was performed using ClinEX pro kit CE-IVD (4 bases, Manno, Switzerland) on NextSeq550dx platform (Illumina, San Diego, CA, USA). Variant filtering and prioritization were performed using the eVAI tool (EnGenome, Pavia, Italy) through a trio-based strategy that included the proband and both parents. Sanger sequencing was used to confirm the segregation of the variant in the healthy family members.

To investigate the effect of the novel *MAOA* splice variant, total RNA was extracted from the patient’s and a healthy control’s leukocytes, followed by cDNA synthesis and sequencing.

### 4.4. Functional Study of the c.955+1G>A

To investigate the effect of the novel identified *MAOA* variant, total RNA was extracted from the patient’s leukocytes and a healthy control. RNA isolation was performed using PAXgene Blood RNA tube (PreAnalytiX-Qiagen, Venlo, The Netherlands) for whole blood collection and RNA stabilization, followed by RNA purification using PAXgene Blood RNA Kit (PreAnalytiX-Qiagen, Venlo, The Netherlands) according to the manufacturer’s instructions. The RNA concentration and purity were determined on a spectrophotometer and stored at −20 °C. cDNA was obtained using High-Capacity cDNA Reverse Transcription Kit (ThermoFisher, Waltham, MA, USA), which is a random primer method allowing cDNA synthesis of all species of RNA molecules present, including mRNA. Primer design was performed using the NCBI RefSeq database for the mRNA reference sequence (NM_000240.4) and the Primer3 tool for primer design. Specificity of the designed primers was assessed using BLASTn v.2.17.0 to ensure the absence of significant complementarity with other mRNA sequences. The region from exon 7 to exon 10 was amplified using the following primers: Exon 7F: 5′–cgctgaaccatgaacattatgag–3′; Exon 10R: 5′–ctttccgggcaagaatgaag–3′.

### 4.5. Variant Effect In Silico Prediction

The potential impact of the identified variant on RNA splicing was evaluated using in silico prediction tools, including SpliceAI v.1.3.1 and the Ensembl Variant Effect Predictor (VEP) v.114.0. In addition, gene expression data from the Genotype-Tissue Expression Project (GTEx) were analyzed to assess tissue-specific expression patterns and to infer the potential impact of nonsense-mediated mRNA decay across tissues.

## 5. Conclusions

In conclusion, the genotype-driven diagnosis enabled the recognition of Brunner syndrome in a patient and guided targeted clinical management, resulting in tangible clinical benefits. These findings further expand the molecular spectrum of *MAOA* variants and highlight the clinical utility of genetic testing in allowing the establishment of a precise molecular diagnosis and supporting precision medicine-oriented clinical management.

## Figures and Tables

**Table 1:** *MAOA* variants associated with clinical, behavioural and biochemical features across published clinical cases of Brunner syndrome.

Case	*Brunner* et al. 1993 [6]	*Piton* et al. 2014 [8]		*Palmer* et al. 2016 [9]		*Minniti* et al. 2024 [10]	*Current Case*
***MAOA*** **variant (NM_000240.4)**	c.886C>T (p.Gln296Ter)	c.797_798delinsTT (p.Cys266Phe)		c.749_750insT (p.Ser251Lysfs*2)	c.133C>T (p.Arg45Trp)	c.410A>G (p.Glu137Gly)	c.955+1G>A
**Predicted effect**	Nonsense variant	Missense variant		Nonsense variant	Missense variant	Missense variant	Affecting splicing site
**Classification**	Pathogenic	Pathogenic		Pathogenic	Likely pathogenic	Uncertain significance	Likely pathogenic
**Patients**	14 affected adult males	2 affected adult males	1 child (7-year-old boy)	2 affected adult males	2 affected adult males; one carrier mother	1 child (5-year-old boy); one carrier mother	24-year-old affected male
**Carrier mother**	Normal intelligence and behaviour	Normal intelligence and behaviour	Normal intelligence and behaviour	Normal intelligence and behaviour	Normal intelligence and behaviour; flushing/diarrhea/headache symptoms	Mild intellectual disability; occasional night terror, palpitation, headache, abdominal pain and swelling	Normal intelligence and behaviour; headache and moderate depression
**Intellectual disability**	Borderline intellectual disability	Variable intellectual disability	Psychomotor delay	Mild intellectual disability	Borderline-mild intellectual disability	Mild intellectual disability	Intellectual disability
**Behavioural characteristics**	Stress-induced impulsive behaviour (aggression, arson, attempted rape, exhibitionism)	Auto- and hetero-aggressive bursts	Aggressive impulse, hand stereotypies and behavioural abnormalities	Impulsive aggressive behaviour	Impulsive aggressive behaviour: ADHD and stereotypical hand movements	Autism spectrum disorder, hetero-aggressive behaviours	Impaired language, social communication, behavioural regulation and stereotyped motor behaviours
**Sleep features**	Night terrors	Not available	Night terrors and frequent awakenings	Difficulties with sleep and wake cycle (with no night terror)	Severe nightmares	Parasomnia	Parasomnia, night terrors and phobic symptoms
**Serotonergic features**	Not available	Not available	Not available	No specific serotonergic symptoms	Flushing/diarrhea/headache (carrier mother included)	No specific serotonergic symptoms	No specific serotonergic symptoms
**Biochemical abnormalities**	High 5-HT and NMN; Low HVA, VMA, 5-HIAA	High NMN and MN; 5-HT not available; Low VMA and 5-HIAA		High 5-HT and NMN; Low VMA	High 5-HT and NMN; Low VMA	High 5-HT and NMN; Low VMA and 5-HIAA	No specific abnormalities
**Treatment**	Not available	Not available	Not available	Not available	SSRIs	SSRIs: carrier mother	SARI

Abbreviations: 5-HIAA, 5-hydroxyindoleacetic acid; 5-HT, 5-hydroxytryptamine or serotonin; HVA, homovanillic acid; MN, metanephrine; NMN, normetanephrine; VMA, vanillylmandelic acid; ADHD, attention-deficit hyperactivity disorder; SSRIs, selective serotonin reuptake inhibitors; SARI, serotonin antagonist and reuptake inhibitor.

## Data Availability

All relevant data are available from the corresponding author upon request.

## Supplementary Materials

The following supporting information can be downloaded at: https://www.mdpi.com/article/10.3390/ijms27146223/s1.
